# Experimental Rhus Poisoning

**Published:** 1914-10

**Authors:** L. B. Bibb

**Affiliations:** Austin, Texas


### Experimental Rhus Poisoning.

WRITTEN FOR TEXAS MEDICAL JOURNAL BY L. B. BIBB, M. D., AUSTIN, TEXAS.

The following case brings out into relief several facts about dermatitis venenata and its cure:

Mr. X, a college instructor, well versed in botany, presented himself for treatment on August 10. He had a lesion on the inner surface of right knee, and consisting of a red base with whitish blisters superposed. The lesion was like the ordinary lesion of poison oak. After several applications of lead water the lesion healed. The patient had the impression that he was entirely insusceptible to poison oak and stated that he knew exactly where he came in contact with the plant that caused the inflammation. It was decided to go out in the woods to the spot where the patient had knelt down on some foliage, and to ascertain what plant was responsible for the damage. This was at least a week after the original exposure and no further lesions had developed.

When the particular part of the woods in question was visited a small stalk of poison oak or rhus not over eight inches high was found in the precise spot where the knee had been pressed against the ground in the act of kneeling and reaching out over a pool of water. The patient being a scientist himself, desired to demonstrate beyond question that he was susceptible to poison oak, and so, by the use of a piece of cardboard with a hole in the center, the poison oak was rubbed vigorously against a sharply delimited area on the arm. The area coming in contact with the plant was one-fourth the size of a postage stamp. A vaccination shield of celluloid, but perforated, was fastened by adhesive over the point of application of the rhus. A second person also supposed to be immune to rhus poisoning was also thus vaccinated.

Within twenty-four hours the characteristic lesions of rhus dermatitis had appeared in various places on both forearms and the left upper arm. The area that has been inoculated with the rhus plant showed as a red raised spot. Grouped around it and below it were dozens of small macules and papules with a few vesicles, having somewhat the appearance of miliaria. The other forearm (right) showed a few dime-sized groups of vesicles pin-head size, and just above the elbow, the vesicles were grouped so as to roughly suggest a band two inches wide running across the lower end of the upper arm. This suggested the offending agent had been spread by the shirt sleeve which had been worn rolled up just above the elbow.

Under treatment by lead water the lesions rapidly disappeared.

The second person inoculated showed absolutely no reaction at any time.

From this case certain deductions can be drawn. It is evident that there is a marked variation in individual susceptibility to rhus poisoning. The two cases in question showed on the one hand absolute immunity, and on the other hand a mild susceptibility. It would be easy to imagine a higher degree of susceptibility, which would give us the sloughing, swelling and other distressing symptoms occasionally seen.

From the band of eruption on the right arm one would suspect that the irritant might be transferred by clothing, utensils and other objects which come in contact with the skin. Franz Pfaff states that his handling of some hundred pounds of the green drug in the laboratory failed to show any evidence of aerial transmission.

The prompt subsidence of the disease under the influence of lead water would indicate that this remedy is of value. The author believes that lead water will destroy all the irritant with which it comes in contact. In a severe case it is well to apply the lead water half a dozen times first; then treat the skin lesion with emollients. In the meantime, an entire change of clothing should be substituted.

